# Author Correction: Dislocation exhaustion and ultra-hardening of nanograined metals by phase transformation at grain boundaries

**DOI:** 10.1038/s41467-022-33775-y

**Published:** 2022-10-07

**Authors:** Shangshu Wu, Zongde Kou, Qingquan Lai, Si Lan, Shyam Swaroop Katnagallu, Horst Hahn, Shabnam Taheriniya, Gerhard Wilde, Herbert Gleiter, Tao Feng

**Affiliations:** 1 Herbert Gleiter Institute of Nanoscience, School of Material Science and Engineering, Nanjing University of Science and Technology, Nanjing, 210094 China; 2 Institute of Nanotechnology, Karlsruhe Institute of Technology, Karlsruhe, 76021 Germany; 3 Institute of Materials Physics, University of Münster, Münster, 48149 Germany; 4 Shenyang National Laboratory for Materials Science, Institute of Metal Research, Chinese Academy of Sciences, Shenyang, 110016 China; 5 Present Address: Key laboratory for Light-Weight Materials, Nanjing Tech University, Nanjing, 211816 China

**Keywords:** Metals and alloys, Structural properties

**Correction to**: *Nature Communications* 10.1038/s41467-022-33257-1, published online 17 September 2022

The original version of this Article contained an error in the Acknowledgements, in which the grant number for National Natural Science Foundation of China was previously incorrectly given as ‘55001166’. The correct version states ‘52001166’ in place of ‘55001166’. This has been corrected in both the PDF and HTML versions of the Article.

