# Supplementary material for: Dislocation exhaustion and ultra-hardening of nanograined metals by phase transformation at grain boundaries
Source: Nat Commun. 2022 Sep 17;13:5468. doi: 10.1038/s41467-022-33257-1 (PMC9482613; doi:10.1038/s41467-022-33257-1)
Supplement: Supplementary file 1 — Supplementary Information [file 41467_2022_33257_MOESM1_ESM.pdf]

Supplementary Information for

Dislocation exhaustion and ultra-hardening of nanograined metals by phase transformation at grain boundaries

Shangshu Wu[1,#], Zongde Kou[1,#], Qingquan Lai[1,2]*, Si Lan[1], Shyam Swaroop Katnagallu[3], Horst Hahn[1,3], Shabnam Taheriniya[4], Gerhard Wilde[1,4], Herbert Gleiter[1,3,5], Tao Feng[1]*

[1] Herbert Gleiter Institute of Nanoscience, School of Material Science and Engineering, Nanjing University of Science and Technology, Nanjing 210094, China.

[2] Now at: Key laboratory for Light-weight Materials, Nanjing Tech University, Nanjing 211816, China.

[3] Institute of Nanotechnology, Karlsruhe Institute of Technology, Karlsruhe 76021, Germany.

[4] Institute of Materials Physics, University of Münster, Münster 48149, Germany.

[5] Shenyang National Laboratory for Materials Science, Institute of Metal Research, Chinese Academy of Sciences, Shenyang, 110016, China.

[#] These authors contributed equally: Shangshu Wu, Zongde Kou.

* email: qingquanlai@hotmail.com; tao.feng@njust.edu.cn

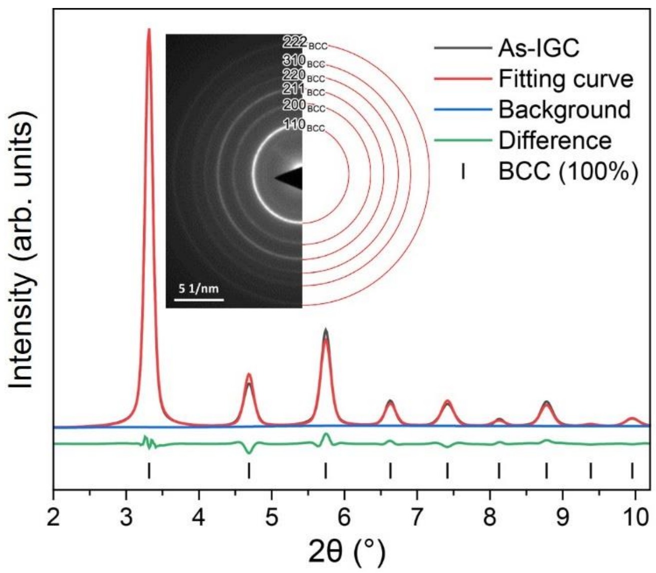

Supplementary Fig. 1 Phase identification of the as-IGC Fe-Ni alloy. The Rietveld refinement result for the as-IGC Fe-Ni alloy shows a single BCC phase, which is confirmed by the inserted selected area electron diffraction (SAED) patterns.

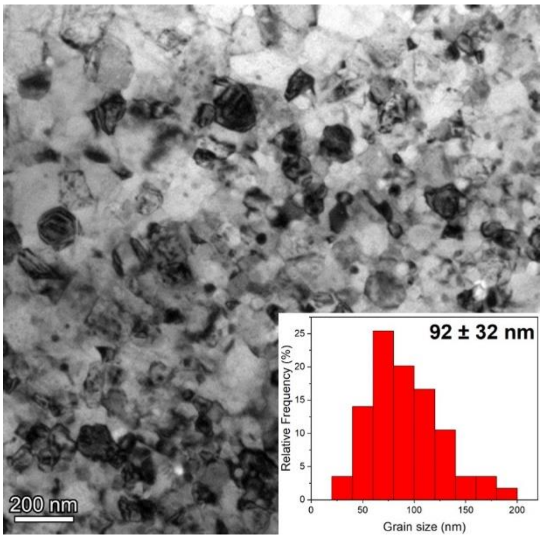

Supplementary Fig. 2 Microstructure of the IGC Fe-Ni alloy annealed at 500 ºC for 1 h. BF TEM image of the IGC Fe-Ni annealed at 500 ºC for 1 h. The inserted histogram shows the grain size distribution.

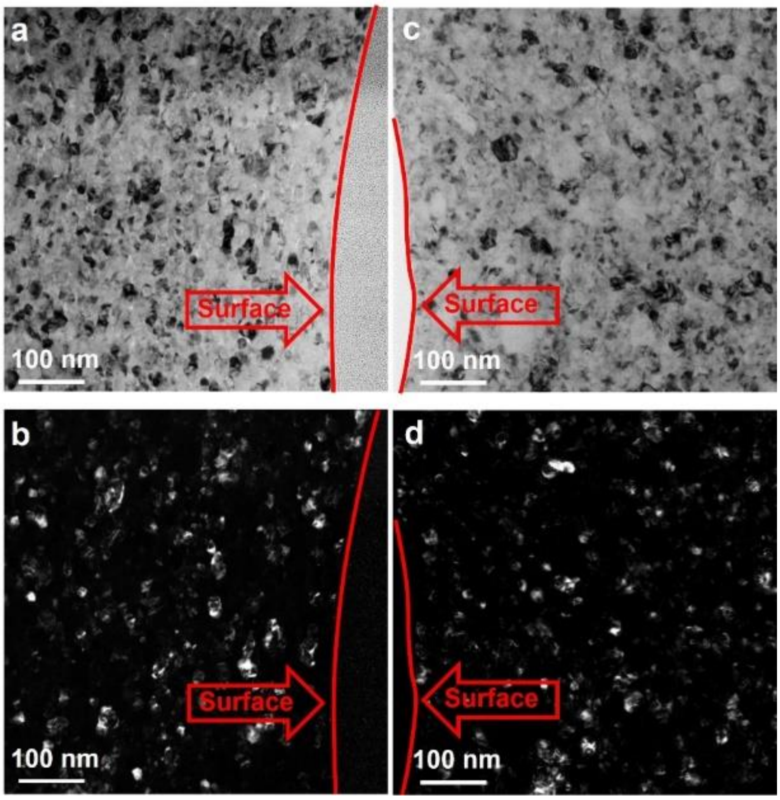

Supplementary Fig. 3 Microstructure underneath the indented surfaces. The BF TEM and DF TEM images of **a-b** the as-IGC and **c-d** annealed (300 ºC for 1 h) Fe-Ni nanograins underneath the indented surface.

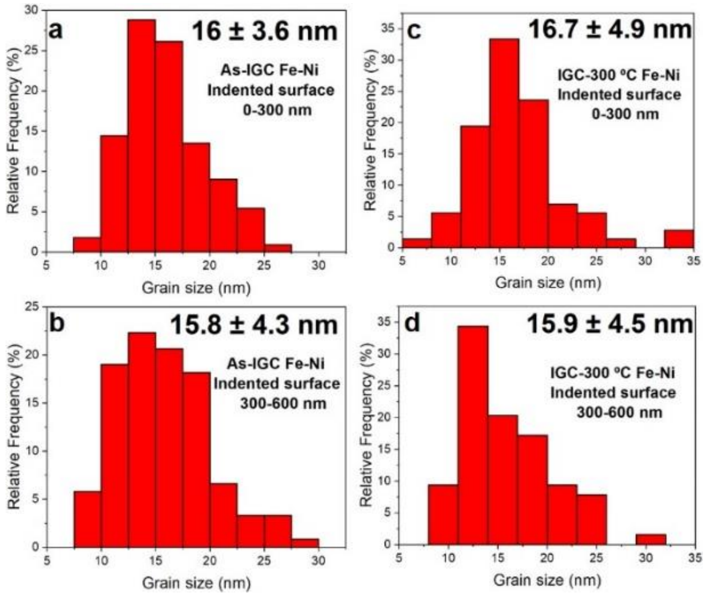

Supplementary Fig. 4 Grain size distribution underneath the indented surfaces. Grain size distribution of the as-IGC Fe-Ni nanograins under the indented surface **a** 0-300 nm and **b** 300-600 nm. Grain size distribution of the annealed (300 ºC for 1 h) Fe-Ni nanograins under the indented surface **c** 0-300 nm and **d** 300-600 nm.

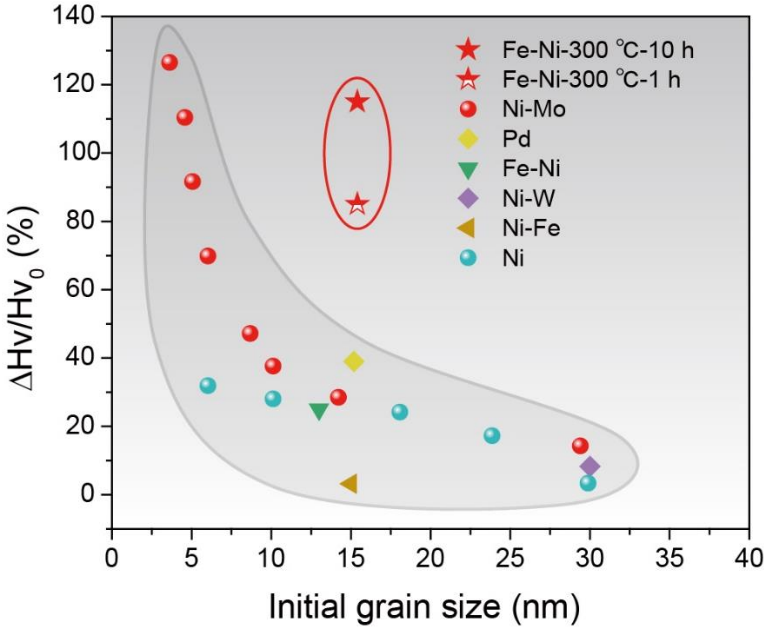

Supplementary Fig. 5 Hardening efficiency of the IGC Fe-Ni alloys. Relationship between the hardening efficiency $\Delta H_V/H_{V0}$ (H_{V0}: initial hardness) induced by annealing and the initial grain size for a variety of alloys (Refs.[1-5]).

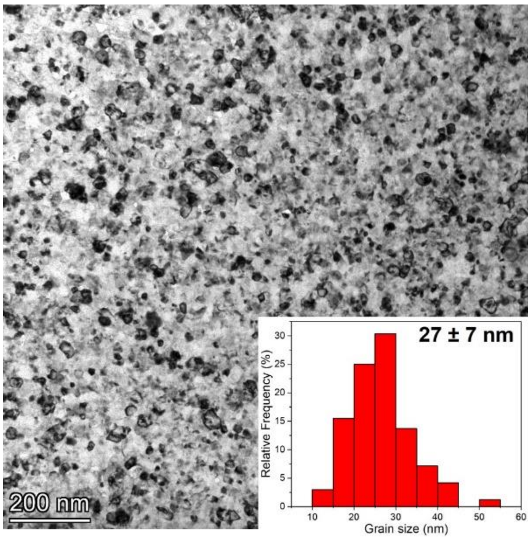

Supplementary Fig. 6 Microstructure of the IGC Fe-Ni alloy annealed at 300 ºC for 10 h. BF TEM image of the IGC Fe-Ni annealed at 300 ºC for 10 h. The inserted histogram shows the grain size distribution.

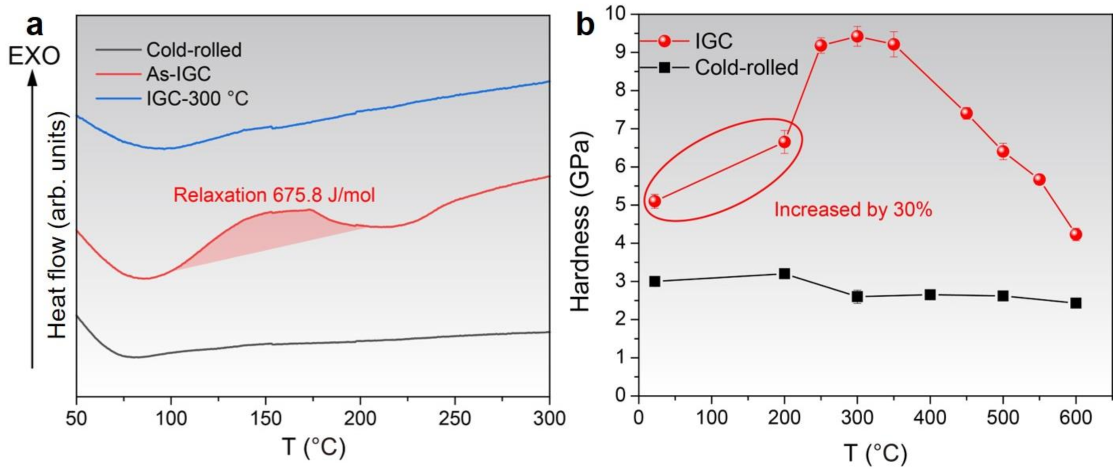

Supplementary Fig. 7 Probing the thermal and mechanical effects of grain boundary relaxation. a shows the DSC curves of the cold-rolled, as-IGC and IGC-annealed Fe-Ni samples. The excess energy of the as-IGC sample is estimated. The hardening effect corresponding to the annealing at this temperature is marked in **b**.

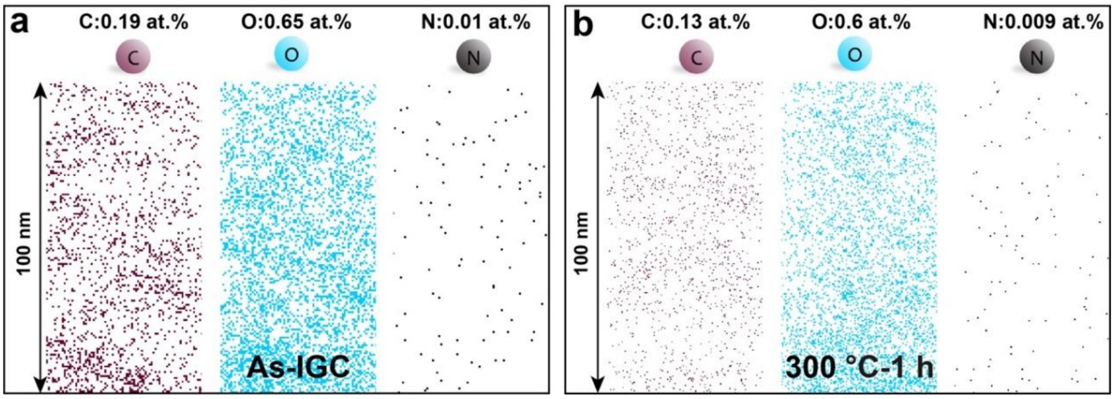

Supplementary Fig. 8 The spatial distribution of the impurities of carbon, oxygen, and nitrogen. The 3D reconstruction of impurities (C, O, N) in Fe-Ni nanograined alloy **a** before and **b** after annealing at 300 ℃ for 1 h.

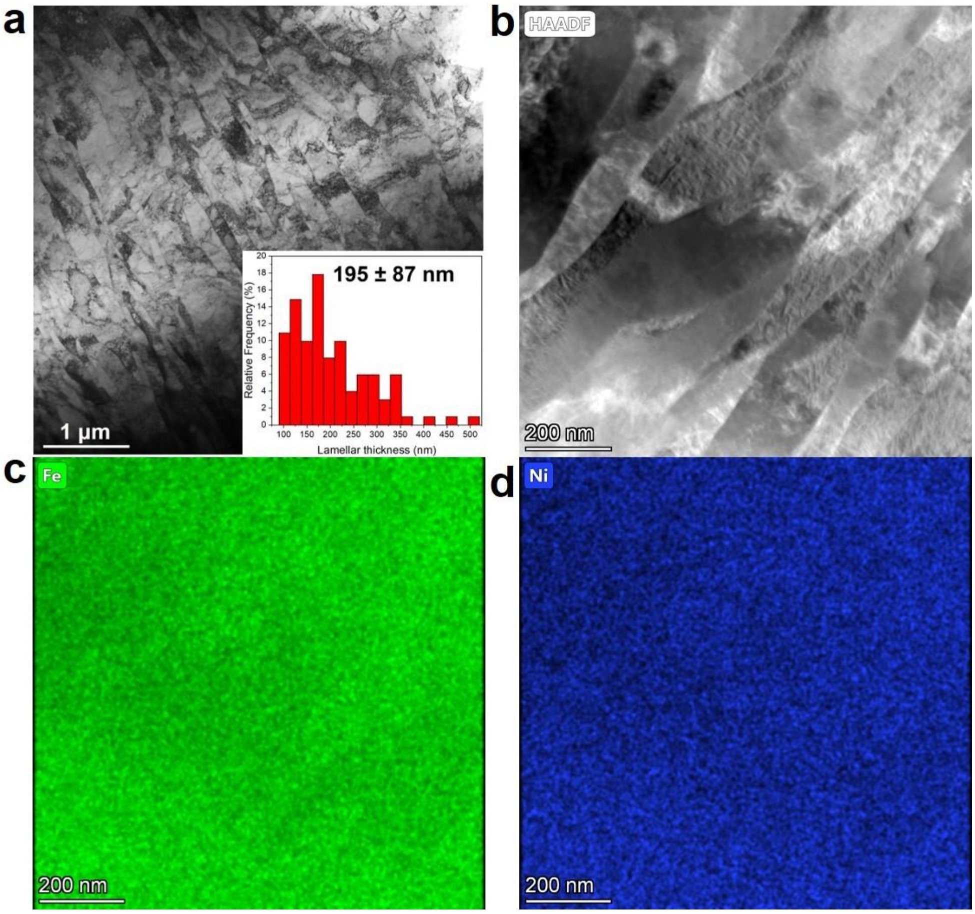

Supplementary Fig. 9 BF TEM image and elemental distributions of the cold-rolled Fe-Ni sample. a BF TEM image of the cold-rolled Fe-Ni sample, the inserted histogram shows the lamellar thickness distribution. **b** HAADF result of the cold-rolled Fe-Ni sample. **c and d** The corresponding STEM-EDX elemental maps of **b**. No segregation can be observed.

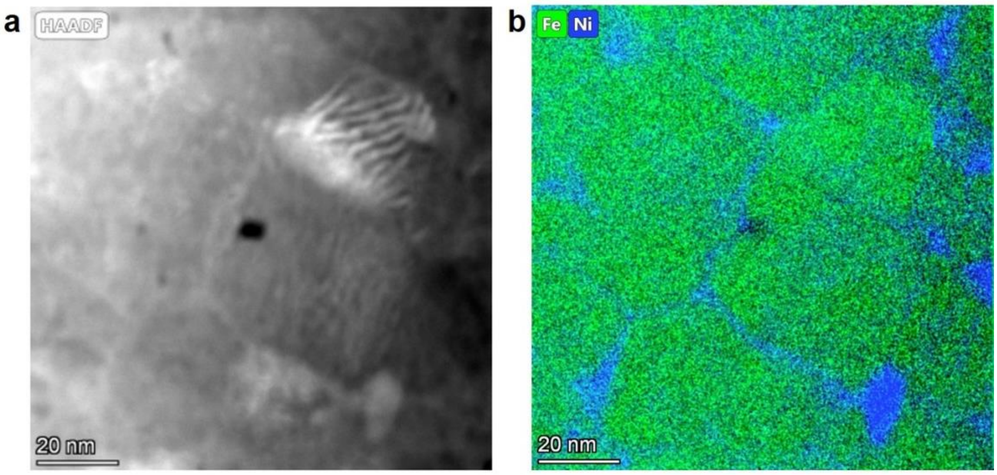

Supplementary Fig. 10 Elemental distributions upon annealing. a HAADF and **b** STEM-EDX elemental maps of IGC-300 ºC-1 h Fe-Ni. Segregation can be observed around grain boundaries and at triple junctions in **b**.

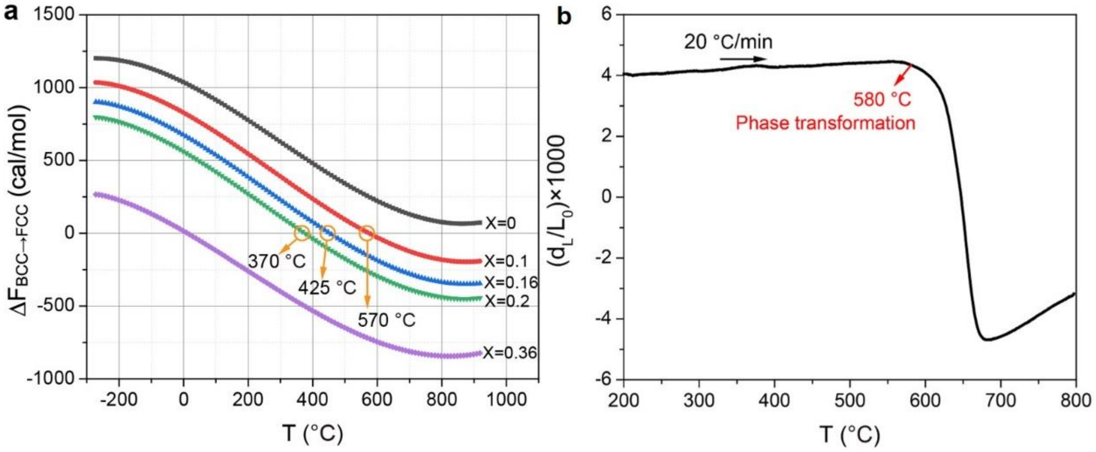

Supplementary Fig. 11 Chemical and physical characteristics of the studied alloy. a Chemical free energy change accompanying the BCC→FCC transformation in the Fe-Ni system (X is the atom fraction of solute Ni in the Fe-Ni alloy). **b** TMA result shows the phase transformation of the cold-rolled Fe-Ni sample occurred at 580 ℃, which is much higher than that of IGC Fe-Ni samples.

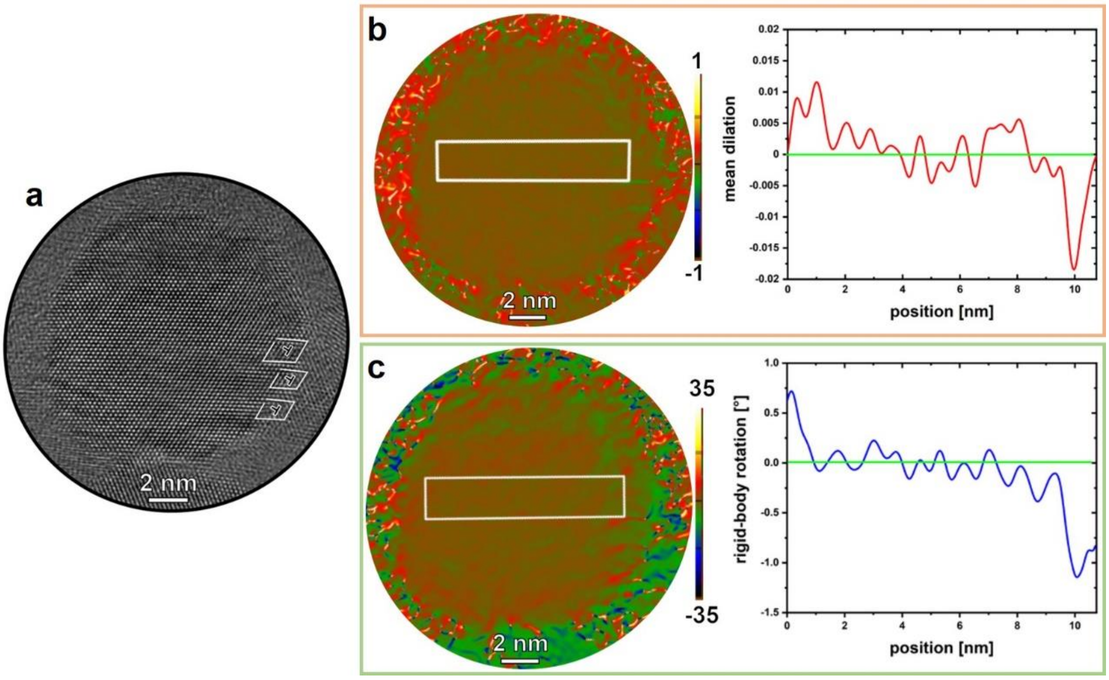

Supplementary Fig. 12 The geometrical phase analysis of an individual nanograin. a HRTEM image of as-IGC Fe-Ni, residual dislocations are detected adjacent to grain boundary. **b** The strain distribution of the mean dilatation in the grain was measured in the form of a profile, where the grain boundary regions show higher values of the strain distribution of the mean dilatation. **c** Rigid-body rotation gradient of the as-IGC Fe-Ni grain is shown by the profiles indicated by the white boxes.

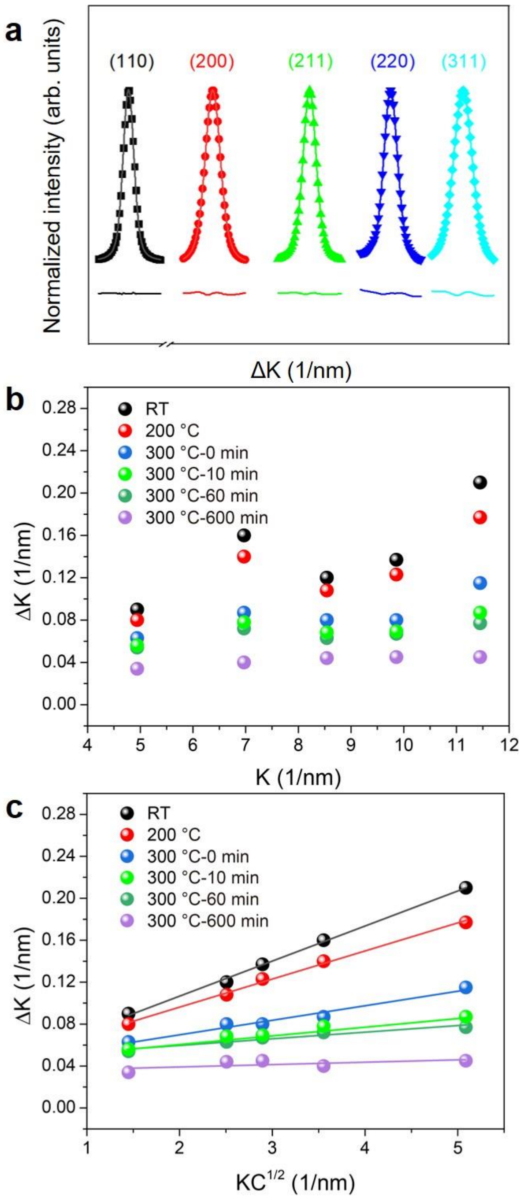

Supplementary Fig. 13 Details of the synchrotron XRD profile analysis. a Normalized experimental intensities with superimposed Gaussian—Cauchy curves fitting to the experimental intensities. In the lower part of the figure, differences between the measured data points and fitted functions are shown. **b** and **c** Plots of ΔK vs. K and $KC^{1/2}$ according to the conventional Williamson–Hall and modified Williamson–Hall, details are shown in the section of calculation of dislocation density.

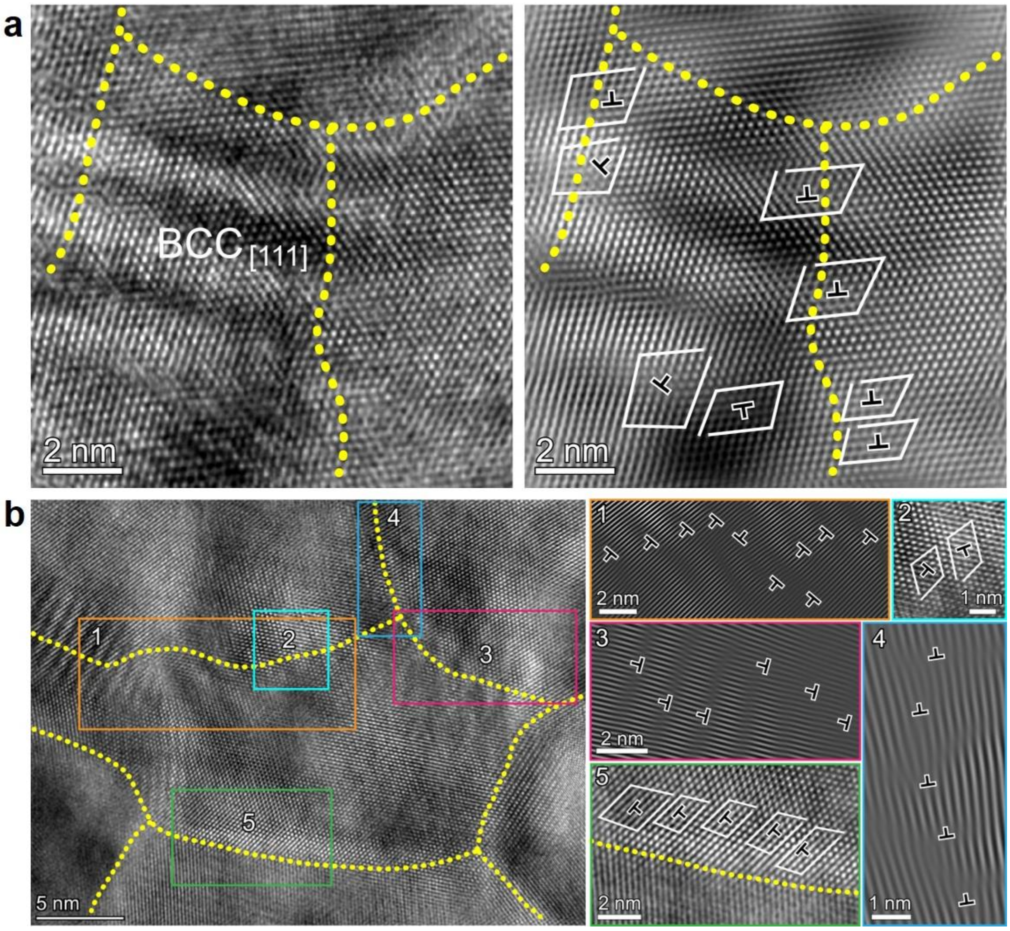

Supplementary Fig. 14 HRTEM observations of the nanograined microstructures. a shows grain boundary region (marked by dotted yellow line) and few grain internal dislocations (marked by T shape symbols) of the as-IGC Fe-Ni. **b** shows the dislocations distribution underneath the indented surface of the IGC-300 ºC Fe-Ni samples.

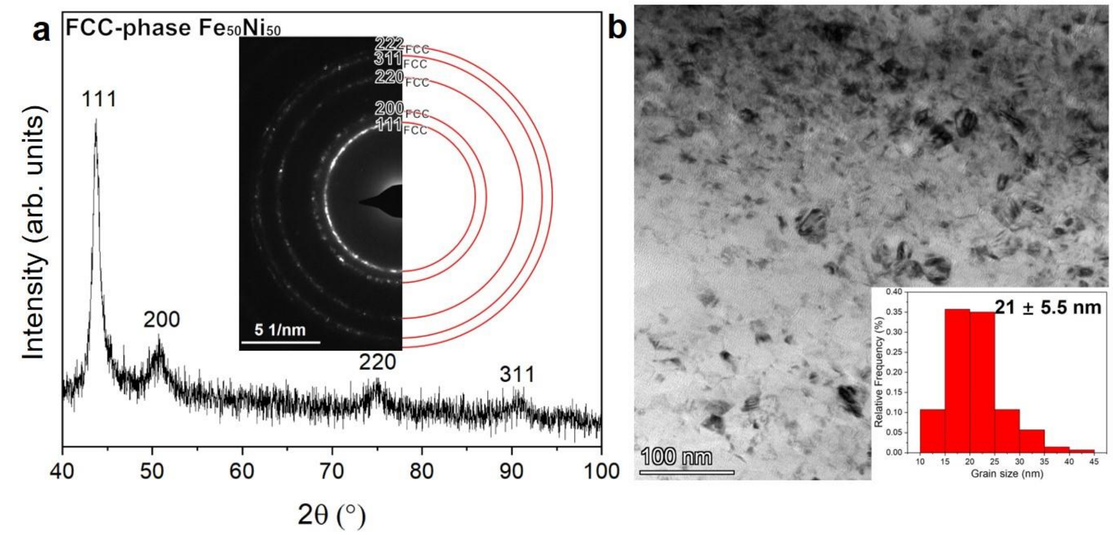

Supplementary Fig. 15 Microstructure of the as-IGC $Fe_{50}Ni_{50}$ alloy. a XRD pattern of the as-IGC $Fe_{50}Ni_{50}$ alloy, it shows single FCC structure, the insert is the SAED result. **b** Bright field TEM image of the as-IGC $Fe_{50}Ni_{50}$ alloy, the inserted histogram shows the grain size distribution.

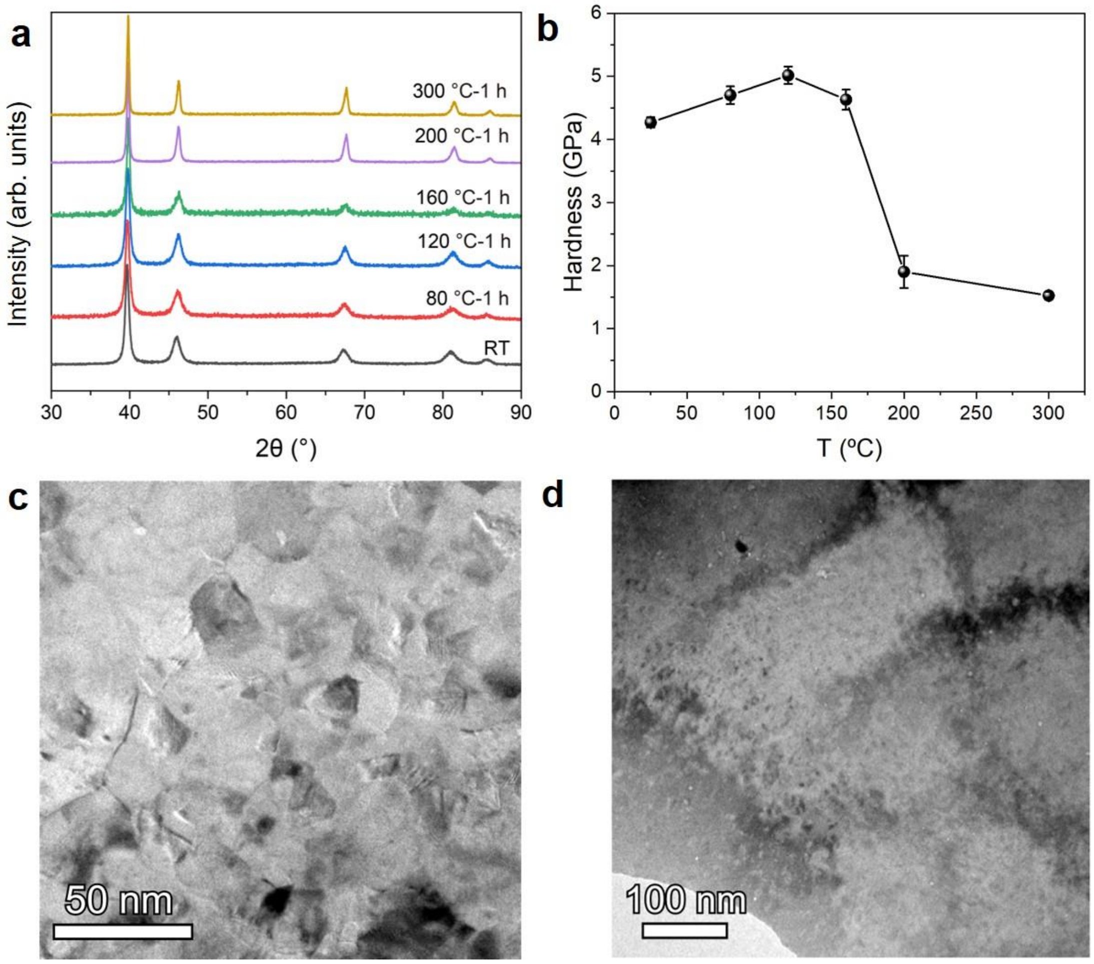

Supplementary Fig. 16 Effect of annealing on the microstructure and hardness of nanograined Pd-Au alloy prepared by IGC. a XRD patterns for the IGC Pd-Au alloy annealed at different temperatures for 1h. **b** Microhardness variations as a function of annealing temperature. **c** and **d** show the BF TEM images of the IGC Pd-Au alloy before and after annealing at 160 ºC, respectively.

Supplementary Table. 1 Variation of dislocation density and hardness during the heat treatment

**Samples**	**Dislocation density (m^{-2})**	**Grain size (TEM) (nm)**	**Dislocation density reduction ratio**	**Hv (GPa)**	**$\Delta H_V/H_{V0}$**
**As-IGC**	1.2×10^{15}	15.4	/	5.1	/
**IGC-200 ºC**	7.5×10^{14}	/	37.5%	6.2	21.8%
**IGC-300 ºC**	2.0×10^{14}	/	83.3%	6.7	31.0%
**IGC-300 ºC-10 min**	5.6×10^{13}	/	95.3%	8.0	56.9%
**IGC-300 ºC-60 min**	4.1×10^{13}	17.0	96.6%	9.4	84.5%

Supplementary References

1. Hu, J., Shi, Y.N., Sauvage, X., Sha, G. & Lu, K. Grain boundary stability governs hardening and softening in extremely fine nanograined metals. *Science* **355**, 1292-1296 (2017).
2. Matsui, I., et al. Enhancement in mechanical properties of bulk nanocrystalline Fe–Ni alloys electrodeposited using propionic acid. *Mater. Sci. Eng. A* **607**, 505-510 (2014).
3. Weertman, J. R., Sanders, P. G. Plastic Deformation of Nanocrystalline Metals. *Solid State Phenom.* **35-36**, 249-262 (1993).
4. Matsui, I., Kanetake, M., Mori, H., Takigawa, Y., Higashi, K. Relationship between grain boundary relaxation strengthening and orientation in electrodeposited bulk nanocrystalline Ni alloys. *Mater. Lett.* **205**, 211-214 (2017).
5. Wang, Y. M., et al. Effects of annealing and impurities on tensile properties of electrodeposited nanocrystalline Ni. *Scr. Mater.* **51**, 1023-1028 (2004).
